# Optical Coherence Tomography Angiography of Macular Perfusion Changes after Anti-VEGF Therapy for Diabetic Macular Edema: A Systematic Review

**DOI:** 10.1155/2021/6634637

**Published:** 2021-05-22

**Authors:** Ayman G. Elnahry, Gehad A. Elnahry

**Affiliations:** ^1^Department of Ophthalmology, Faculty of Medicine, Cairo University, Cairo, Egypt; ^2^Elnahry Eye Clinics, Giza, Egypt

## Abstract

**Background:**

Diabetic macular edema (DME) is a major cause of vision loss in diabetics that is currently mainly treated by antivascular endothelial growth factor (VEGF) agents. The effect of these agents on macular perfusion (MP) is a current concern. Optical coherence tomography angiography (OCTA) is an imaging modality that allows noninvasive high-resolution retinal microvasculature imaging. Several recent studies evaluated the effect of anti-VEGF agents on the MP of DME patients using OCTA. Our aim is to provide a systematic review of these studies.

**Methods:**

Multiple databases were searched including PubMed, Ovid Medline, EMBASE, and Google Scholar for relevant studies published between January 2016 and November 2020 which were included in this review. Studies were compared regarding their design, the number of included patients, the machine and scanning protocol used, the inclusion and exclusion criteria, the number of injections given, the type of anti-VEGF agent used, the outcome measures assessed, and the effect of injections on different MP parameters.

**Results:**

A total of 16 studies were included. The studies assessed various OCTA parameters that define MP including the foveal avascular zone area and superficial and deep vascular density and yielded conflicting results. Seven studies showed stable or improved MP following treatment, while 7 studies showed worsening MP following treatment, and 2 studies showed inconclusive results. This could have been due to differences in study design, inclusion criteria, type of anti-VEGF agents used, treatment duration, and methods of image analysis and vascular density quantification. All identified studies were noncomparative case series, and 14 of them (87.5%) used the RTVue XR Avanti OCTA machine. Only one study compared OCTA to fluorescein angiography findings.

**Conclusion:**

Analysis of MP changes following VEGF inhibition for DME could benefit from a unified scanning protocol and analysis approach that uses similar study designs to eliminate potential sources of bias. This may provide more definitive conclusions regarding the effect of treatment on MP.

## 1. Introduction

**Figure 1:**
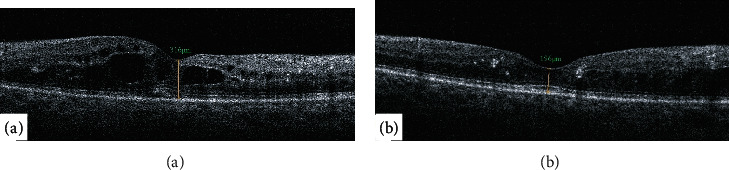
Optical coherence tomography (OCT) of the macula of a diabetic patient showing improvement of DME before (a) and after (b) anti-VEGF injections. There is marked improvement in the central foveal thickness as measured by OCT.

Diabetic macular edema (DME) is the most common cause of loss of vision in diabetic patients, affecting around 21 million people globally [1]. The treatment of macular edema associated with various retinal diseases, including DME (Figure 1), is currently dependent on the repeated intravitreal injection of different anti-vascular endothelial growth factor (anti-VEGF) agents [2–6]. Other treatment modalities for DME include steroid injections and macular laser photocoagulation [2].

VEGF-A is one of the members of the VEGF family and is currently the most implicated in the pathogenesis of these various retinal conditions due to its role in angiogenesis and vaso-permeability which results in the disruption of blood-retinal barriers [2, 7]. It has 5 different isoforms, and most of the currently available anti-VEGF antibodies block all these isoforms [2]. VEGF has also several physiologic functions that include the regulation of normal vasculogenesis and angiogenesis [8]. It also acts as a survival factor for retinal vessels during their development [9], maintains the choriocapillaris and the photoreceptors [10], and possesses a neuroprotective role that reduces neuronal apoptosis [11]. These physiological functions of VEGF may be interrupted during prolonged treatment of retinal conditions with repeated injection of anti-VEGF agents, which raises concerns regarding the safety of the long-term use of these agents [12, 13].

**Figure 2:**
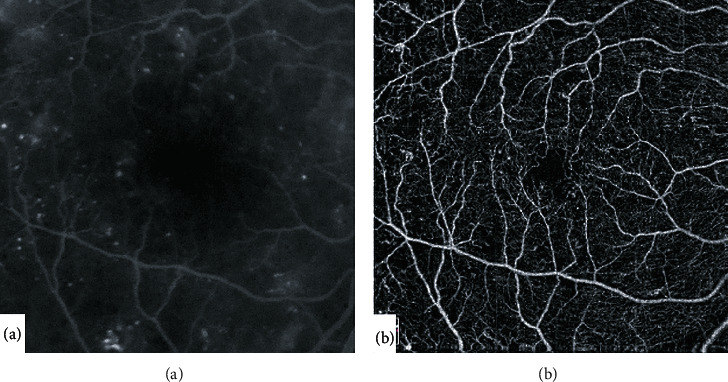
Compared to fluorescein angiography (a), optical coherence tomography angiography (Optovue, Inc., Fremont, CA, USA) of the macula (b) allows imaging of the retinal capillaries and foveal avascular zone in high resolution without obscuration by dye leakage or macular xanthophyll pigment shadowing.

Several animal studies have shown deleterious cellular effects following the inhibition of VEGF [10, 14, 15]. A number of previous studies, however, have evaluated the effect of repeated intravitreal injections of VEGF inhibitors for DME on the macular and retinal perfusion of diabetic patients using fluorescein angiography (FA) with some studies showing worsening and others showing no worsening of perfusion following treatment. These studies, however, assessed mainly the superficial retinal vasculature, were possibly influenced by dye leakage, and depended on trained human graders [16–22]. Optical coherence tomography angiography (OCTA) allows noninvasive and dye-free imaging of the superficial and deep retinal vasculature providing high resolution 3D images of the different retinal vascular layers separately [23, 24]. OCTA has been previously shown to precisely and reliably delineate areas of capillary drop-out and to image the foveal avascular zone (FAZ) without obscuration by dye leakage or macular xanthophyll pigment shadowing compared to FA (Figure 2) [24–26]. It also allows quantitative and automated measurements of the retinal vascular density (VD) and the fractal dimension (FD) reliably and in a reproducible manner in the macular area without the need for human graders compared to FA [27–31].

Recently, several studies have been performed by various investigators using OCTA to objectively evaluate the effect of repeated intravitreal anti-VEGF injections on the macular perfusion of diabetic patients with DME. In this systematic review, we aim to summarize and compare the methods and findings of these studies.

## 2. Methods

### 2.1. Registration, Search Strategy, and Database Search

This systematic review was prospectively registered at PROSPERO on November 25, 2020 (Registration number: CRD42020216343). The study followed the tenets of the Declaration of Helsinki and was approved by the Cairo University research ethics committee. The Preferred Reporting Items for Systematic Reviews and Meta-analyses (PRISMA) guidelines were followed. A systematic literature search using PubMed, Ovid MEDLINE, EMBASE, Science Direct, and Google Scholar for articles published between January 2016 and November 2020 was performed. Keywords searched included different combinations of the following terms: diabetic macular edema, diabetic macular ischemia, diabetic retinopathy, anti-VEGF, VEGF inhibition, ranibizumab, aflibercept, bevacizumab, macular perfusion, macular ischemia, macular microvasculature, retinal ischemia, retinal perfusion, perifoveal capillaries, foveal avascular zone, capillary reperfusion, capillary non-perfusion, vascular density, and optical coherence tomography angiography. For example, the search strategy in PubMed was as follows: “diabetic macular edema” AND “anti-VEGF OR bevacizumab OR ranibizumab OR aflibercept” AND “macular perfusion OR macular microvasculature OR foveal avascular zone OR diabetic macular ischemia OR vascular density” AND “optical coherence tomography angiography”.

### 2.2. Selection of Studies

Following the exclusion of duplicates, the identified titles and abstracts were independently reviewed by both investigators, and all relevant studies were included in the review. Any disagreement between both investigators was resolved by an open discussion. The reference lists of identified papers were also examined to find additional relevant articles.

### 2.3. Inclusion and Exclusion Criteria

Studies in which OCTA was performed before and after anti-VEGF injections for the treatment of DME were included. Studies which did not perform OCTA before injections and studies which only used FA were excluded. Studies which used steroid injections for treatment of DME were also excluded. Only articles that were peer-reviewed and published in the English language were included, and no restrictions were applied to the study type.

### 2.4. Data Extraction and Assessment of Methodological Quality and Risk of Bias

All included studies were read in full independently by both investigators, and the following data was extracted: name of authors, title, year of publication, the design of the studies, the number of included patients, the scanning protocol and type of machine used, the inclusion and exclusion criteria of patients, the number of injections given and the treatment protocol used, the type of anti-VEGF agents used (Bevacizumab (Avastin, Genentech/Roche, South San Francisco, CA, USA), aflibercept (Eylea, Regeneron, Tarrytown, NY, USA), ranibizumab (Lucentis, Genentech/Roche, South San Francisco, CA, USA), and conbercept (Lumitin, Chengdu Kanghong Biotech, Sichuan, People's Republic of China)), the outcome measures assessed, and the effect of the injections on different macular perfusion parameters. Data extracted by both investigators was compared, and any discrepancies were discussed, and a consensus was reached. The assessment of the methodological quality and risk of bias in and across the included studies was performed using a customized scoring scale as follows: eight items were scored for each study according to whether they were present or absent. The presence of an item amounted to a score of one while its absence amounted to a score of zero. The maximum score possible was 8, and a higher score meant a higher study quality.

## 3. Results

**Figure 3:**
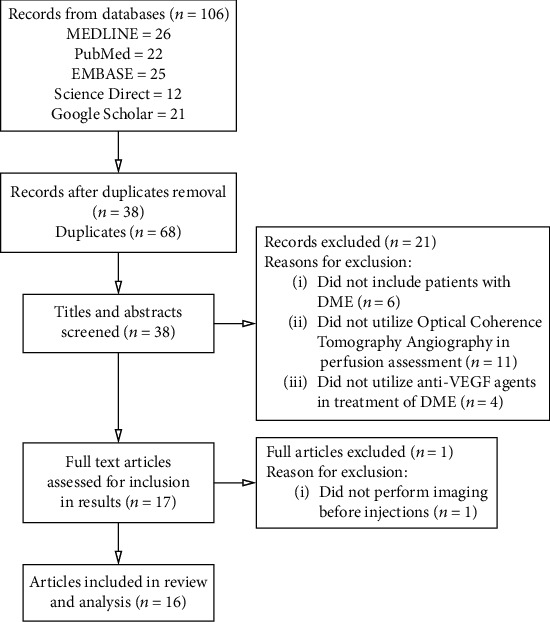
Flow diagram showing search results and reasons for exclusion of studies.

A total of 16 studies that evaluated the effect of intravitreal anti-VEGF injections for DME on the macular perfusion using OCTA in the period between January 2016 and November 2020 were included [12, 32–46].  Figure 3 shows the PRISMA flowchart summarizing the results of the search strategy and reasons for exclusion. All identified studies were noncomparative case series, and so, the Jadad Scale, the Newcastle-Ottawa Scale, and the Cochrane Collaboration's tool could not be used to assess or compare their methodological quality or risk of bias.

Most of the included studies (14/16, 87.5%) used the RTVue XR Avanti Spectral domain-OCTA machine (Optovue, Inc., Fremont, CA, USA), while two studies (2/16, 12.5%) used the Triton OCTA machine (Topcon Inc, Japan). Only one study compared ischemic to nonischemic eyes [37], while one study compared OCTA to FA findings [41]. The duration of treatment and type of anti-VEGF agent used were variable, and some studies utilized multiple anti-VEGF agents. The design, methods, and results of the included studies are summarized in Tables 1, 2, and 3 according to whether positive, negative, or conflicting effects were found on the macular perfusion, respectively, following treatment.  Table 4 summarizes the strengths and limitations of all included studies, while Table 5 presents the customized scale for assessing and comparing the methodological quality of the included studies.

### 3.1. Studies That Found Stable or Improved Macular Perfusion following Injections (Table 1)

A total of 7 studies found stable or improved macular perfusion using OCTA following anti-VEGF injections for DME. In the study by Ghasemi Falavarjani et al. [32], OCTA was prospectively used to evaluate the macular perfusion changes following a single injection of VEGF inhibitors in patients with macular edema due to either DR or central retinal vein occlusion (CRVO). The authors found that neither the FAZ area nor the foveal and parafoveal vascular density of both the superficial (SCP) and deep capillary plexuses (DCP) changed significantly after the single injection. Further evaluation of data from the study showed that the FAZ area increased, and the VD of the foveal area decreased following the single injection, which was not, however, statistically significant. The study, however, included a relatively small number of eyes, used various types of anti-VEGF (bevacizumab in 14 eyes, aflibercept in 3 eyes, and ranibizumab in 1 eye), included 2 different etiologies for the macular edema (13 eyes with DME and 5 eyes with CRVO), involved a single intravitreal injection, had a limited follow-up of one month, and used VD measurements from the built-in machine software. Patients with a history of previous anti-VEGF treatment were also not excluded.

Sorour et al. [33] retrospectively evaluated the effect of repeated injections of VEGF inhibitors for DME or proliferative diabetic retinopathy (PDR) on the macular perfusion using OCTA and found no statistically significant difference in VD measurements after 1, 2, and 3 injections using two scanning protocols. The study included 55 eyes: 46 underwent OCTA imaging after the 1st injection, 28 after the 2nd injection, and 26 after the 3rd injection. Multiple anti-VEGF agents were also used (45.7% bevacizumab, 42.4% aflibercept, and 11.9% ranibizumab) with a mean interval of 47 days between the injections. 23 eyes (50%) that performed OCTA scanning after the 1st injection were not treatment naïve: 13 eyes were included after a washout period of 3 months from the last anti-VEGF injection, while 10 eyes were on regular VEGF inhibitors treatment without a washout period.

In the retrospective study by Hsieh et al. [34], a custom-developed Matlab (Mathworks, Natick, MA, USA) software was used for OCTA image processing and analysis. Five OCTA biomarkers including FAZ area, FAZ circulatory index, average vessel caliber, vessel tortuosity, and VD were assessed before and after 3 monthly ranibizumab injections for DME. The authors reported a statistically significant improvement in the FAZ (-31%), the average vessel caliber (-4.3%), and the inner (+6%) and outer (+9%) parafovea VD of the SCP and a statistically significant improvement in the FAZ (-31%), the FAZ circulatory index (-4.2%), and the inner (+9%) and outer (+9.4%) parafovea VD of the DCP following the injections. These biomarkers, however, did not improve to their normal values as compared to a healthy control group. The study did not find significant correlations between OCTA metrics and anatomical improvement but the inner parafoveal VD of the SCP turned out to be the biomarker that most correlated with visual improvement after treatment.

In the retrospective study by Conti et al., patients with DR that underwent aflibercept injections for DME were included, and the FAZ area and retinal VD were measured at baseline and after 6 and 12 months of treatment [35]. No patient was treatment naïve (average of 5.1 injections before the study with an average washout period of 44.4 days), and by 12 months, 26% received monthly aflibercept injections while 74% received bimonthly injections with an average of 5.2 injections before switching to bimonthly. Although an enlargement of the FAZ area from 0.307 mm^2^ to 0.313 mm^2^ and decrease in whole image VD from 46.9% to 45.7% was reported at 12 months, these changes were not statistically significant (*p* > 0.05). Changes in choriocapillaris density which was also evaluated by OCTA were also not statistically significant.

Michalska-Małecka and Heinke Knudsen retrospectively reviewed patients imaged before and after treatment with 3 to 5 injections of aflibercept for DME using OCTA [36]. The authors reported little change in the VD measurements of the patients, but found an improvement of vision and central macular thickness.

In a prospective study by Zhu et al. [37], the effect of conbercept injections on the macular perfusion of patients with DME was assessed using OCTA scanning. Patients were divided into two subgroups: ischemic (31 patients) and nonischemic (19 patients) according to macular perfusion findings on FA and received 3 monthly conbercept injections followed by pro re nata regimen for another 3 months. The FAZ area showed a significant decrease in the ischemic group, while the VD of the SCP increased. Measurements in the nonischemic group did not significantly change.

Mirshani et al. [38] prospectively evaluated changes in macular perfusion following a single bevacizumab injection for DME using OCTA. They measured the retinal VD, FAZ area, vessel diameter index, vascular length density, capillary nonperfusion area, and subfoveal choriocapillaris VD before and after the injection. No significant changes were found after the injection except for a mild improvement in choriocapillaris VD. Only patients with a recent history of VEGF inhibitor treatment were excluded.

### 3.2. Studies That Found Worsening of Macular Perfusion following Injections (Table 2)

A total of 7 studies found worsening of macular perfusion following ant-VEGF injections for DME using OCTA. In the observational study by Couturier et al. [39], patients with DME and severe nonproliferative or proliferative DR injected with ranibizumab or aflibercept for 3 months were included in the study and assessed using OCTA. Following the injections, there was a decrease in the VD of the SCP from 39.5 ± 6.9% to 36.6 ± 4.3% and of the DCP from 44.7 ± 6.2% to 42.5 ± 3.8%. The result, however, was not statistically significant.

Our group performed a prospective study aimed at evaluating by OCTA the effects of 3 monthly intravitreal injections of bevacizumab on the macular perfusion of treatment naïve diabetic patients with DME (the IMPACT Study) [40]. The FAZ area enlarged by 8.1%, the full retinal thickness (Full) and the SCP FD was reduced by 1.3%, the FD of DCP was reduced by 1.9%, the VD of Full was reduced by 8%, the VD of the SCP was reduced by 9.1%, the VD of DCP was reduced by 10.6%, the skeleton VD of Full was reduced by 13.3%, the skeleton VD of the SCP was reduced by 12.5%, and the skeleton VD of the DCP was reduced by 16.3% in the 6 × 6 mm macular area imaged by OCTA following the injections which were all statistically significant (*p* < 0.05) [40]. Similar findings were also found in the 3 × 3 mm scanning protocol. Automated image alignment before and after treatment was performed using a retinal alignment software (i2k Align Retina, DualAlign, LLC, Clifton Park, NY, USA), and segmentation errors were corrected manually.

In a small prospective study by Pereira et al. that included patients with DME associated with moderate to severe macular ischemia, the functional and anatomical effects of bevacizumab were assessed using fluorescein angiography, OCTA, and microperimetry [41]. Following 6 intravitreal injections, the mean area of the FAZ on fluorescein angiography was reduced from 1.35 ± 1.44 mm^2^ to 1.02 ± 1.02 mm^2^ (*p* = 0.19) although 3 eyes exhibited an enlargement of the FAZ area. The mean FAZ area on OCTA (3 eyes analyzed) enlarged from 0.82 ± 0.55 mm^2^ to 0.92 ± 0.57 mm^2^ (*p* = 0.02). Microperimetry showed improvement of macular sensitivity from 11.66 ± 0.77 dB to 16.26 ± 3.29 dB (*p* = 0.007) which better correlated with improvement in retinal thickness than with ischemic areas on either fluorescein angiography or OCTA. Three of the 5 included eyes in that study were previously treated with anti-VEGF agents while 2 eyes were treatment-naïve.

In a prospective long-term study conducted by our group, 2 eyes of a diabetic patient sequentially treated with repeated monthly bevacizumab injections for DME showed decreased VD measurements of the SCP and DCP following the injections using OCTA. These measurements subsequently returned to their baseline value when the injections were withheld [12]. Treatment of the left eye with injections led to reversible decrease in the VD while the VD of the untreated right eye was stable for 7 months without injections. Following treatment of the right eye there was a similar decrease in the VD.

In the retrospective study by Barash et al., macular and peripapillary VD changes were evaluated immediately following an intravitreal injection of a VEGF inhibitor (bevacizumab or aflibercept) for different pathologies including DR which showed a statistically significant reduction in the VD of most areas of the SCP and DCP in association with elevation of the intraocular pressure, with particularly more affection in the temporal SCP, the optic nerve head, and the radial peripapillary capillary network [42]. The superficial VD was reduced by 8%, while the deep VD was reduced by 3.5% immediately (within 3 minutes) after the injection.

In a prospective, interventional, single-arm study of patients with persistent DME, Statler et al. found a significant decrease in the superficial and deep capillary perfusion density, including the whole, foveal, and parafoveal density, following fixed interval intravitreal injections of aflibercept for 24 months [43]. Better vision correlated with less loss of capillary perfusion density following the injections in the superficial whole and parafoveal areas. Only 16 patients completed the study. All participants had a history of VEGF inhibitor treatment before inclusion in the study.

Golshani et al. [44] prospectively evaluated patients with DME that switched from bevacizumab (95%) or ranibizumab (5%) to aflibercept using OCT and OCTA (SWAP-TWO Study). Patients received monthly aflibercept injections until OCT demonstrated no fluid, followed by fixed dosing once every two months through 12 months. Following treatment, there was no significant change in the FAZ area, but the VD of the SCP significantly decreased by 5.2% (*p* = 0.04), and the VD of the DCP significantly decreased by 6.3% (*p* = 0.05) by 12 months.

### 3.3. Studies That Found Conflicting Effects following Treatment (Table 3)

In the retrospective study by Busch et al. [45] which analyzed the FAZ area and the VD following repeated aflibercept injections (mean: 2.6 ± 1.3 injections) for DME using OCTA. The authors reported an enlargement of the FAZ area in the SCP from 0.41 ± 0.2 mm^2^ to 0.48 ± 0.24 mm^2^ but a reduction of the FAZ area in the DCP from 0.75 ± 0.34 mm^2^ to 0.71 ± 0.33 mm^2^. These changes, however, were not statistically significant. The VD measurements were also unchanged following the injections.

Dastiridou et al. [46] used swept source OCTA to evaluate the FAZ and VD measurements of eyes of treatment naïve patients with DME following 3 aflibercept injections. There was a statistically significant reduction in the size of the FAZ of the DCP but not of the SCP following the injections with a high intragrader and intergrader agreement for the manual FAZ measurements. The VD of SCP of the central macular area showed a statistically significant 8% reduction following the injections, whereas the decrease in parafoveal VD did not reach statistical significance. The authors hypothesized that the displacement of the capillaries by the macular edema and the associated segmentation errors which occurred and were restored following treatment may have played a role in the findings of their study.

## 4. Discussion

The effects of repeated intravitreal injections of different VEGF inhibitors for DME on the macular perfusion of diabetic patients has been recently evaluated in several studies that used the relatively new OCTA technology. Originally, this evaluation has depended on the use of FA and expert human graders [16–22], with several studies that later followed that employed ultra widefield FA imaging in order to evaluate the effect of these VEGF inhibitors on the peripheral retinal perfusion of diabetics as well [39, 47–49]. These studies, however, did not provide conclusive results regarding the effect of VEGF inhibition on the status of the macular perfusion of diabetic patients [50].

OCTA is a dye-free imaging modality that depends on comparison of the decorrelation signals between repeated consecutive OCT B-scans acquired in rapid succession at the same retinal location. This allows motion contrast generated by the flow of red blood cells in the retinal vessels to be detected which leads to imaging of perfused retinal vessels and detection of flow-void areas [23–26, 51]. OCTA, therefore, makes it possible to obtain reliable and reproducible measurements of VD and FD, thus allowing an objective and grader-independent assessment of the macular perfusion status [27–31]. Based on these features, OCTA harbors great potential in DR evaluation and is probably more suitable than traditional angiography in analyzing VD changes following VEGF inhibition in diabetics. Nevertheless, OCTA is a still emerging technology with several limitations. First of all, OCTA images may be significantly impacted by low signal strength, resulting in an altered visualization of the small vessels. Moreover, localized loss of signals due to localized media opacities can be misinterpreted as flow-void areas. Finally, it should be considered that vascular OCTA imaging is currently more affected by artifacts and interpretation errors compared to FA. These artifacts include motion and blink artifacts (due to the prolonged scanning time), shadow artifacts, and projection artifacts from superficial layers [52, 53]. The interpretation of OCTA images should, therefore, be done cautiously with these limitations in mind to avoid reaching misleading or inaccurate conclusions. This could possibly explain why results of studies performed by different investigating groups using OCTA may be different or conflicting. However, as the OCTA technology gets better and faster, many of these limitations and artifacts could be eliminated leading to more reliable and comparable results from studies with more solid conclusions.

A relatively large number of studies that utilized OCTA in the evaluation of macular perfusion changes in diabetics following VEGF inhibition have been recently conducted by several research groups globally. This may reflect the current uncertainty that surrounds the effect of VEGF inhibitors on the macular perfusion status of diabetic patients, which led many researchers to attempt to investigate this effect using the emerging OCTA technology considering its advantages in imaging the retinal vasculature over FA imaging that include its ability to image fine capillary details, especially the perifoveal capillary network and deep capillary plexus, in a higher resolution without obscuration by leakage of dye or macular xanthophyll pigment which allows better detection and quantification of retinal ischemia [54–57]. The studies analyzed in this review, however, yielded conflicting results with 7 studies showing stable or improved macular perfusion following treatment [32–38], 7 studies showing worsening of macular perfusion following treatment [12, 39–44], and 2 studies showing conflicting results [45, 46]. This could have been due to study design differences, differences in patients' characteristics and inclusion criteria, or methods of image analysis and vascular density quantification. The relative weight of each study and its overall significance in our opinion depended on several factors including the study design, the number of included patients, the duration of treatment and number of injections given, the treatment protocol used, and the method of image analysis, which could not be assessed with any available scale since all the identified studies were noncomparative case series. We therefore developed a customized scoring scale consisting of these items to help compare the methodological quality and risk of bias across the identified studies based on a validated item bank on the risk of bias and precision of case series [58].

Some factors associated with anti-VEGF treatment for DME may result in macular perfusion improvement while others may lead to its worsening, which could explain why some diabetic patients experience macular perfusion improvement following the injections while others experience perfusion worsening, probably depending on which of these factors predominate in each patient. Even in the single patient, it is apparent that some macular areas become better perfused while other areas become worse on OCTA following treatment which suggests that several factors associated with VEGF inhibition that influence macular perfusion are in play [59]. Factors that could result in retinal perfusion improvement after treatment with anti-VEGF antibodies include the reversal of leukostasis, that is induced by excessive VEGF secretion in diabetics and results in increased capillary occlusion [60], restoration of the normal retinal architecture due to decreased intraretinal edema [46], and inhibition of the hypertrophy of endothelial cells that is induced by excessive local VEGF-A production and leads to narrowing and occlusion of capillary lumen [61]. Factors that could justify retinal perfusion worsening following VEGF inhibition include inducing vasoconstriction of the retinal vasculature which was found following bevacizumab and ranibizumab injections for DME possibly due to nitric oxide inhibition which occurs with VEGF inhibition and also leads to systemic hypertension in case of systemic VEGF inhibition [62, 63]. Inhibition of VEGF by bevacizumab also resulted in a decrease of the mean blood flow velocity of the central retinal, the temporal posterior ciliary, and the ophthalmic artery by about 10%, 20%, and 20%, respectively, 4 weeks after one bevacizumab injection in patients with exudative age-related macular degeneration [64]. Loss of pericytes that normally surround mature retinal capillaries and make them nondependent on VEGF for survival could be another cause of decreased capillary density following VEGF inhibition in diabetics [65, 66]. Loss of pericytes is known to occur in early DR and may render capillary endothelial cells susceptible to VEGF inhibition leading to endothelial cell demise and subsequent capillary loss [14, 67]. Another possibility that could also explain the worsening of macular perfusion associated with VEGF inhibition for DME treatment is the progressive natural history of DR which in this case is incompletely arrested by VEGF inhibition as has been previously reported [21].

The presence of cystoid spaces in chronic diabetic macular edema may resemble areas of capillary nonperfusion on enface OCTA since both may appear as black or grey areas [68, 69]. The appearance of these areas, however, can be variable depending on which OCTA machine is used [69, 70]. Cystoid spaces seen on OCT were also found to colocalize with areas of nonperfusion on OCTA [68, 70]. It is possible that these cystoid spaces may result in lateral displacement of capillaries or preferentially occur in areas of capillary nonperfusion due to the development of nearby leaky microaneurysms [56, 68]. The disappearance of these cystoid spaces following treatment with resultant capillary reperfusion may be another mechanism for an increase in vascular density following treatment [70]. In one study analyzing the effect of treatment on these spaces, however, no reperfusion occurred in nonperfusion areas following resolution of the cystoid spaces [68]. This, however, may have been due to the chronic nature of edema in these cases which requires further validation in cases with the earlier stages of the disease.

The short- and long-term consequences of macular perfusion changes following VEGF inhibition are also still unclear since most of the analyzed studies reported significant visual and anatomical improvements following the injections regardless of the macular perfusion changes. In the study by Hsieh et al. which showed improvement of the macular perfusion following 3 monthly ranibizumab injections, there were no significant correlations between OCTA biomarkers and anatomical improvement; however, the inner parafoveal vascular density of the superficial capillary plexus was the most significantly correlating biomarker with visual improvement following treatment [34], while in the prospective study performed by our group which showed worsening of macular perfusion following 3 monthly bevacizumab injections, changes in the superficial capillary plexus showed a significant negative correlation with changes in the central macular thickness [40]. Other studies, however, did not report significant correlations between OCTA biomarkers and visual or anatomical improvement, with Pereira et al. showing that macular sensitivity measured using microperimetry better correlated with changes in retinal thickness than with ischemic areas on either FA or OCTA [41]. These correlations are important in order to determine the clinical significance of these macular perfusion changes on the short- and long-term functional and anatomical treatment outcomes.

In an interesting study by Alagorie et al. [71] that evaluated the association of intravitreal aflibercept injections with macular VD changes using OCTA in patients with proliferative diabetic retinopathy but without DME, the authors reported no significant macular VD changes following 12 months of intravitreal aflibercept therapy using both monthly and quarterly dosing. The study used the 3 × 3 mm OCTA, however, which may have missed ischemic changes in the perifovea, and only diabetic patients without DME were included since the authors thought that DME may result in artifacts and segmentation errors in OCTA images which they indicated may have affected the results of previous studies; however, the structure and integrity of the macular microvasculature may differ between patients with and without DME, and so, the direct extrapolation of their results to patients with DME may not be valid. For example, patients with DME may have more structural damage to their macular microvasculature in the form of more pericyte loss which may render their vessels more susceptible to VEGF inhibition compared to patients without DME [40, 65]. Also, because the authors did not compare the baseline macular VD values of included patients with a healthy control group, it is difficult to estimate the initial severity of macular ischemia in the included patients. This is important since patients with more macular ischemia at baseline could be at more risk of worsening of ischemia following VEGF inhibition [72]. Finally, although patients in that study did not have initial macular thickening, there was a decrease in the central macular thickness following treatment below what would be considered a normal macular thickness, raising concerns for progressive macular atrophy associated with long-term VEGF inhibition even in the absence of detectable macular perfusion worsening.

## 5. Conclusion

In conclusion, several studies have been performed to evaluate the effect of intravitreal injections of various VEGF blocking agents for DME on the macular perfusion status of diabetics using OCTA yielding conflicting results that could have been influenced by study design, patients' inclusion criteria, and method of image analysis used. Analysis of vascular density changes following anti-VEGF treatment for DME using OCTA could benefit from a unified scanning protocol and analysis approach that uses similar study designs and patients' inclusion criteria to eliminate potential sources of bias. This could ultimately provide more definitive conclusions regarding the effect of these injections on the macular perfusion status of diabetics. With future advances in the OCTA technology, including increased speed of scanning, development of better imaging artifacts correction software, and wider scanning protocols, this evaluation will be more reliable and reproducible.

## Figures and Tables

**Table 1:** Study design, methods, and results of studies that found stable or improved macular perfusion following injections.

Study	No. of eyes	Design of study	Imaging modality	Agent used and treatment duration	Outcome measure	Study results	Ref.
Ghasemi Falavarjani et al.	13 eyes of 10 patients with DME	Prospective noncomparative case series	OCTA (Optovue) using VD from machine software	Bevacizumab, Ranibizumab, Aflibercept (1 injection by any)	Change in FAZ area (manual) and VD	FAZ-A increased, and VD of foveal area decreased but nonsignificantly (*p*>0.05)	[32]
Sorour et al.	55 eyes of 35 patients with DME or PDR	Retrospective case series	OCTA (Optovue) 3 × 3 and 6 × 6 scans with VD of machine	45.7% Bevacizumab, 42.4% Aflibercept, and 11.9% Ranibizumab	Change in VD after 3 injections	No significant change in VD measurements at 1, 2, and 3 months	[33]
Hsieh et al.	50 eyes of 50 patients with DME	Retrospective case series	OCTA (Optovue) 3 × 3 with custom developed Matlab software for image processing and analysis	Ranibizumab (3 injections)	Change in FAZ-A, FAZ-CI, AVC, vascular tortuosity, and VD	Improvement of FAZ-A (-31%), AVC (-4.3%), and inner (+5.9%) and outer (+8.8%) PF-VD in the SCP, and FAZ-A (-31%), FAZ-CI (-4.2%), and inner (+9.1%) and outer (+9.4%) PF-VD in DCP (*p* ≤ 0.05)	[34]
Conti et al.	19 eyes of 19 patients with DR	Retrospective case series	OCTA (Optovue) 6 × 6 scan using built-in machine VD	Aflibercept (by 12 months, 26% received monthly while 74% received bimonthly treatment)	Change in FAZ and VD after 6 and 12 months of treatment	FAZ area enlarged from 0.307 to 0.313 mm^2^, VD dropped from 46.9% to 45.7% (*p* > 0.05)	[35]
Michalska and Heinke	3 eyes of 3 patients with DME	Retrospective case series	OCTA (Optovue) 3 × 3 scans	Aflibercept (3-5 injections)	Change in built-in machine VD	Insignificant change in VD (*p* > 0.05)	[36]
Zhu et al.	50 eyes of 50 patients with DME (ischemic and nonischemic)	Prospective case series with predefined outcome measures	OCTA (Optovue) 6 × 6 scan using machine software	Conbercept (3 monthly injections then pro re nata for 3 months)	Change in built-in machine VD and FAZ area	FAZ area significantly decreased and superficial capillary plexus VD increased in ischemic group (both *p* < 0.05)	[37]
Mirshani et al.	23 eyes of 19 patients with DME	Prospective case series with predefined outcome measures	OCTA (Optovue) 3 × 3 scan using machine software and custom image processing	Bevacizumab (single injection)	Change in built-in machine VD, manual FAZ area, VDI, and VLD index	No significant change in FAZ area, retinal VD, VDI, or VLD (all *p* > 0.05). Improved subfoveal choriocapillaris VD (*p* > 0.05)	[38]

AVC: average vessel caliber; DCP: deep capillary plexus; DME: diabetic macular edema; DR: diabetic retinopathy; FA: fluorescein angiography; FAZ: foveal avascular zone; FAZ-A: foveal avascular zone area; FAZ-CI: foveal avascular zone circulatory index; OCTA: optical coherence tomography angiography; PDR: proliferative diabetic retinopathy; PF: parafoveal; SCP: superficial capillary plexus; VD: vascular density; VDI: vessel diameter index; VLD: vascular length density.

**Table 2:** Study design, methods, and results of studies that found worsening of macular perfusion following injections.

Study	No. of eyes	Design of study	Imaging modality	Agent used and treatment duration	Outcome measure	Study results	Ref.
Couturier et al.	10 eyes of 9 patients with DME	Prospective observational case series	OCTA (Optovue) 3 × 3 scan using machine software for VD	Ranibizumab or Aflibercept (3 injections)	Change in VD after 3 injections using built-in machine software	SCP VD drop from 39.5 ± 6.9% to 36.6 ± 4.3% and DCP VD from 44.7 ± 6.2% to 42.5 ± 3.8% (*p* > 0.05)	[39]
Elnahry et al. (the IMPACT study)	40 eyes of 26 patients with DME	Prospective case series with predefined outcome measures	OCTA (Optovue) 3 × 3 and 6 × 6 scans with custom image processing	Bevacizumab (3 monthly injections)	Change in FD, VD, skeleton VD, and manual FAZ area	Increase in FAZ area and decrease in FD, VD, and skeleton VD of full, SCP, and DCP (all *p* < 0.05)	[40]
Pereira et al.	5 eyes of 5 patients with DME and DMI	Prospective case series	FA, OCTA (Topcon) 3 × 3 or 4.5 × 4.5, and MP	Bevacizumab (6 injections)	Change in FAZ area on FA and OCTA (manually measured in both)	FA FAZ from 1.35 ± 1.44 mm^2^ to 1.02 ± 1.02 mm^2^ (*p* = 0.19) and OCTA FAZ from 0.82 ± 0.55 mm^2^ to 0.92 ± 0.57 mm^2^ (*p* = 0.02)	[41]
Elnahry et al.	2 eyes of 1 patient with DME	Prospective longitudinal case report	OCTA (Optovue) 6 × 6 scan with machine software	Bevacizumab (repeated 3 monthly injections)	Change in built-in machine VD	Reversible worsening of VD with injections	[12]
Barash et al.	9 eyes with PDR and 5 with DME	Retrospective case series	OCTA (Optovue) macular and peri-papillary scans	Bevacizumab or Aflibercept (immediately after injections)	Macular and peri-papillary VD changes	SCP VD dropped by 7.8% while the DCP VD dropped by 3.5% immediately after injection	[42]
Statler et al.	16 eyes of 16 patients with persistent DME	Prospective case series with predefined outcome measures	OCTA (Optovue) 3 × 3 scan using machine software	Aflibercept (fixed interval injections for 24 months)	Change in built-in machine VD and FAZ area	Whole SCP VD decreased by 5.3% and whole DCP VD decreased by 4.4%. FAZ area increased (all *p* < 0.05)	[43]
Golshani et al. (SWAP-TWO study)	20 eyes of 20 patients with DME	Prospective case series with predefined outcome measures	OCTA (Optovue) 3 × 3 scan using machine software	Aflibercept (monthly dosing till dry then every 2 months thereafter through 12 months)	Change in built-in machine VD and FAZ area	No change in FAZ area, but SCP and DCP VD significantly decreased by 5.2% and 6.3%, respectively (*p* ≤ 0.05)	[44]

DCP: deep capillary plexus; DME: diabetic macular edema; DMI: diabetic macular ischemia; FA: fluorescein angiography; FAZ: foveal avascular zone; FD: fractal dimension; Full: full retinal thickness; MP: microperimetry; OCTA: optical coherence tomography angiography; PDR: proliferative diabetic retinopathy; SCP: superficial capillary plexus; VD: vascular density.

**Table 3:** Study design, methods, and results of studies that found conflicting effects following treatment.

Study	No. of eyes	Design of study	Imaging modality	Agent used and treatment duration	Outcome measure	Study results	Ref.
Busch et al.	23 eyes of 23 patients with DME	Retrospective case series	OCTA (Optovue) 3 × 3 scan using machine software for VD	Aflibercept (1-8 monthly injections)	Change in built-in machine VD and FAZ	SCP FAZ increased by 0.07 mm^2^ and DCP FAZ decreased by 0.04 mm^2^ (*p* > 0.05)	[45]
Dastiridou et al.	20 eyes of 20 patients with DME	Prospective case series	Swept source OCTA (Topcon) 6 × 6 and 7 × 7 scans	Aflibercept (3 injections)	Change in FAZ area and VD	FAZ of DCP and VD of central area decreased (*p* ≤ 0.05)	[46]

DCP: deep capillary plexus; DME: diabetic macular edema; FAZ: foveal avascular zone; OCTA: optical coherence tomography angiography; SCP: superficial capillary plexus; VD: vascular density.

**Table 4:** Relative strengths and limitations of identified studies.

Study	Strengths	Limitations	Ref.
Ghasemi Falavarjani et al.	Prospective.	Small number of eyes, used various types of VEGF inhibitors, included 2 different etiologies for macular edema, short duration of treatment, used built-in machine VD measurements, and did not exclude patients previously treated with anti-VEGF.	[32]
Sorour et al.	Relatively large number of eyes and used both 3 × 3 and 6 × 6 scans.	Retrospective, short follow-up period, variable anti-VEGF agent used, variable injection interval, used built-in machine VD measurements, did not exclude patients previously treated with anti-VEGF.	[33]
Hsieh et al.	Relatively large number of eyes, used a custom developed software, used one anti-VEGF agent, included one eye of each patient, assessed multiple outcome measures.	Retrospective, used only 3 × 3 scans and did not use automated image alignment.	[34]
Conti et al.	Used one anti-VEGF agent, had a long duration of follow-up, assessed two treatment protocols.	Retrospective, small number of eyes, used only 6 × 6 scans, used built-in machine VD measurements, did not exclude patients previously treated with anti-VEGF.	[35]
Michalska and Heinke	Used one anti-VEGF agent, included one eye of each patient.	Retrospective, small number of eyes, used only 3 × 3 scans, used variable number of injections, used built-in machine VD measurements.	[36]
Zhu et al.	Prospective, divided patients into ischemic and nonischemic groups, included one eye of each patient, used one anti-VEGF agent.	Used only 6 × 6 scans, did not use automated image alignment, used built-in machine VD measurements.	[37]
Mirshani et al.	Prospective, used one anti-VEGF agent, assessed multiple outcome measures.	Small number of eyes, short follow-up period, used only 3 × 3 scans, included both eyes of some patients, did not exclude patients previously treated with anti-VEGF.	[38]
Couturier et al.	Prospective.	Small number of eyes, included both eyes of some patients, used only 3 × 3 scans, used 2 anti-VEGF agents, used built-in machine VD measurements.	[39]
Elnahry et al. (the IMPACT study)	Prospective, registered, relatively large number of eyes, automated image alignment, used a custom developed software, used both 3 × 3 and 6 × 6 scans, used one anti-VEGF agent, patients were treatment-naïve, assessed multiple outcome measures.	Included both eyes of some patients and short follow-up period.	[40]
Pereira et al.	Prospective, relatively long follow-up period, used a single anti-VEGF agent, used microperimetry and fluorescein angiography.	Small number of eyes, assessed FAZ only, not all eyes were treatment naïve.	[41]
Elnahry et al.	Prospective, fellow eye used as control, long duration of follow-up, treatment naïve patient.	Small number of eyes, used built-in machine VD measurements.	[12]
Barash et al.	Only study to assess effect on VD immediately after the injection.	Retrospective, small number of eyes, short duration of follow-up, variable etiologies included, used built-in machine VD measurements, used 2 anti-VEGF agents.	[42]
Statler et al.	Prospective, long follow-up period, used a single anti-VEGF agent.	Small number of eyes, used only 3 × 3 scans, used built-in machine VD measurements, did not exclude patients previously treated with anti-VEGF.	[43]
Golshani et al. (SWAP-TWO study)	Prospective, long follow-up period, used a single anti-VEGF agent.	Small number of eyes, used only 3 × 3 scans, used built-in machine VD measurements, did not exclude patients previously treated with anti-VEGF.	[44]
Busch et al.	Used a single anti-VEGF agent, included one eye of each patient, patients were treatment naive.	Retrospective, small number of eyes, used only 3 × 3 scans, variable follow-up period, used built-in machine VD measurements.	[45]
Dastiridou et al.	Prospective, used a single anti-VEGF agent, included one eye of each patient.	Small number of eyes, relatively short follow-up period, used built-in machine VD measurements and assessed SCP VD only.	[46]

FAZ: foveal avascular zone; SCP: superficial capillary plexus; VD: vascular density; VEGF: vascular endothelial growth factor.

**Table 5:** A customized scale for assessing and comparing the quality of included studies.

Study	Prospective	More than 30 eyes included	Single anti-VEGF agent	Single eye of included patient	Both 3 × 3 and 6 × 6 scans used	Treatment naive	Follow-up more than 3 months	Customized VD assessment	**Total score**	Ref.
Ghasemi Falavarjani et al.	1	0	0	0	0	0	0	0	**1**	[32]
Sorour et al.	0	1	0	0	1	0	0	0	**2**	[33]
Hsieh et al.	0	1	1	1	0	0	0	1	**4**	[34]
Conti et al.	0	0	1	1	0	0	1	0	**3**	[35]
Michalska and Heinke	0	0	1	1	0	0	0	0	**2**	[36]
Zhu et al.	1	1	1	1	0	0	1	0	**5**	[37]
Mirshani et al.	1	0	1	0	0	0	0	1	**3**	[38]
Couturier et al.	1	0	0	0	0	1	0	0	**2**	[39]
Elnahry et al. (the IMPACT Study)	1	1	1	0	1	1	0	1	**6**	[40]
Pereira et al.	1	0	1	1	0	0	1	0	**4**	[41]
Elnahry et al.	1	0	1	0	0	1	1	0	**4**	[12]
Barash et al.	0	0	0	1	0	0	0	0	**1**	[42]
Statler et al.	1	0	1	1	0	0	1	0	**4**	[43]
Golshani et al. (SWAP-TWO study)	1	0	1	1	0	0	1	0	**4**	[44]
Busch et al.	0	0	1	1	0	1	1	0	**4**	[45]
Dastiridou et al.	1	0	1	1	0	0	0	0	**3**	[46]

The presence of an item amounted to a score of one while its absence amounted to a score of zero. Eight items were scored for each study; thus, the maximum score possible for a study was 8. A higher score meant a higher quality.

## Data Availability

All the data used in this study are available within the article.

## Abbreviations

AVC: Average vessel caliber
CRVO: Central retinal vein occlusion
DCP: Deep capillary plexus
DM: Diabetes mellitus
DME: Diabetic macular edema
DR: Diabetic retinopathy
DRSS: Diabetic retinopathy severity score
ETDRS: Early Treatment Diabetic Retinopathy Study
FA: Fluorescein angiography
FAZ: Foveal avascular zone
FAZ-A: Foveal avascular zone area
FAZ-CI: Foveal avascular zone circulatory index
FD: Fractal dimension
Full: Full retinal thickness
IOP: Intraocular pressure
MP: Microperimetry
OCT: Optical coherence tomography
OCTA: Optical coherence tomography angiography
PDR: Proliferative diabetic retinopathy
PF: Parafoveal
SCP: Superficial capillary plexus
VD: Vascular density
VDI: Vessel diameter index
VLD: Vascular length density
VEGF: Vascular endothelial growth factor.

## Additional Points

The authors have read the PRISMA 2009 Checklist, and the manuscript has been prepared and revised according to its guidelines.

## Supplementary Materials

Supplementary Materials PRISMA 2009 guideline checklist.
